# Special Issue: “Role of MicroRNA in Cancer Development and Treatment”

**DOI:** 10.3390/jpm12030503

**Published:** 2022-03-21

**Authors:** Alessandra Pulliero, Alberto Izzotti

**Affiliations:** 1 Department of Health Sciences, University of Genoa, Via Pastore 1, 16132 Genoa, Italy; 2 Department of Experimental Medicine, University of Genova, 16132 Genova, Italy; izzotti@unige.it; 3 UOC Mutagenesis and Cancer Prevention, IRCCS Ospedale Policlinico San Martino, 16132 Genova, Italy

Exposure to environmental contaminants may lead to changes in the expression of microRNAs (miRNAs), resulting in several health effects. miRNAs, small non-coding RNAs that regulate gene expression, have multiple transcript targets and thereby regulate several signaling molecules. Altered patterns of miRNAs can be responsible for changes linked to various health outcomes, suggesting that specific miRNAs are activated in pathophysiological processes. Genome-wide profiling demonstrates that miRNA expression signatures are associated with tumor type, tumor grade, and clinical outcomes, so miRNAs are potential candidates for diagnostic biomarkers, prognostic biomarkers, therapeutic targets, and preventive screening programs. Although miRNAs have multiple targets, their function in tumorigenesis is due to their regulation of a few specific targets. This Special Issue, entitled “Role of MicroRNA in Cancer Development and Treatment”, focuses on the current state of pharmacogenomics and the extensive translational process required for clinical implementation, including the impact of environmental exposure on the MicroRNA machinery and cancer development. The present Special Issue provides a comprehensive overview of the current status of this interesting field of research. It comprises manuscripts reporting novel data as well as state-of-the-art reviews. The issue begins with five articles [1,2,3,4] focus on the role of microRNAs in environmental risk factors and lung cancer development.

Exposure to asbestos can cause cancer and other health conditions. A rare and aggressive cancer called mesothelioma is almost exclusively caused by asbestos exposure. Asbestos also causes a progressive lung disease called asbestosis. Environmental exposure to asbestos has been associated with a higher incidence of malignant mesothelioma. This study aimed to validate the predicted diagnostic significance of hsa-miR-323a-3p, hsa-miR-101-3p, and hsa-miR-20b-5p on a subset of mesothelioma patients exposed to asbestos and matched with healthy controls. PCR results showed that the three analyzed miRNAs were significantly down-regulated in cases vs. controls. In silico results showed a potential prognostic role of hsa-miR-101-3p due to a significant association of its higher expression and increased overall survival of mesothelioma patients [5]. Malignant mesothelioma is characterized by poor prognosis and short survival. Extracellular vesicles were isolated from serum samples obtained before and after treatment using ultracentrifugation on 20% sucrose cushion. Serum EV-enriched miR-103-3p, miR-126-3p and miR-625-3p were quantified using qPCR. After treatment, the expression of miR-625-3p and miR-126-3p significantly increased in mesothelioma patients with poor treatment outcome. Bioinformatics analysis showed enrichment of 33 miR-625-3p targets in eight biological pathways [6]. A review examines the role of microRNAs, the expression profile of which changes upon exposure to asbestos, in key processes of carcinogenesis, such as proliferation, cell survival, metastasis, neo-angiogenesis, and immune response avoidance [1]. As exposure to air pollution represents a dominant factor in the development of lung cancer and other respiratory system disorders, the authors identified the miRNAs commonly affected by both conditions. Such molecules could serve as biomarkers of choice for identifying human populations at greater risk of lung cancer resulting from exposure to air pollution. The literature search identified a total of 25 miRNAs that meet such criteria. Among them, miR-222, miR-21, miR-126-3p, miR-155, and miR-425 may be considered the prominent molecules, as they were identified to be deregulated in multiple studies [3]. The knowledge of the mechanisms of action of environmental pollution now includes not only the alteration of the gut microbiota but also the interaction between different human microbiota niches such as the lung–gut axis. The epigenetic regulations can reprogram differentiated cells in response to environmental changes. In subjects at high risk of cancer, gut and lung microbiota are distinct from those of low-risk subjects, and disease progression is associated with microbiota alterations [4]. Next in this Special Issue are two contributions describing the relationship between miRNA profiles and oncogene mutations in non-smoker lung cancer and the identification, by microRNA analysis, of environmental risk factors using integrated DNA adducts and microRNAs analyses to retrospectively study the contribution of exposures to environmental carcinogens to lung cancer in 64 non-smokers living in Sicily and Catania city near to the Etna volcano [7]. MicroRNAs play a role in silencing mutated oncogenes, thus defending the cell against the adverse consequences of genotoxic damages induced by environmental pollutants. In addition, certain environmental compounds (i.e., diesel, ozone, and UV radiation) have been identified as persistent environmental pollutants due to their indestructible chemical and physical properties [8], in an experimental study, provide new information on the novel mechanisms on microRNA alteration in human skin biopsies exposed to diesel fumes, ozone, and UV light over 24 h of exposure. UV and ozone induced microRNA alteration immediately after exposure, whereas the peak of their deregulations induced by diesel fumes was reached only at the end of the 24 h. Diesel fumes mainly altered microRNAs involved in the carcinogenesis process, ozone in apoptosis, and UV in DNA repair. Epigenetics includes the study of the hypothesis that exposure to plasticizers causes changes in or the deregulation of a number of oncogenic miRNAs, and shows that the interaction of plasticizers with several redundant miRNAs, such as let-7f, let-7g, miR-125b, miR-134, miR-146a, miR-22, miR-192, miR-222, miR-26a, miR-26b, miR-27b, miR-296, miR-324, miR-335, miR-122, miR-23b, miR-200, miR-29a, and miR-21, might induce significant alterations. This systematic review points out the fact that the altered expression of microRNAs plays an important pathogenic role in exposure to plasticizers [9].

Consequently, human epigenetic studies have explored the identification and validation of miR-210 and miR-152 as non-invasive circulating biomarkers for the diagnosis and staging of breast cancer patients, confirming their involvement in tumor angiogenesis [10].

The expression of miRNAs was analyzed in primary tumors, metastases, and in bone marrow infiltrates of therapy-responsive and non-responsive neuroblastoma patients, in order to identify specific miRNAs involved in neuroblastoma metastasization and chemo-resistance identifying miRNAs involved in the regulation of drug response and employed for therapeutic purposes [11]. Glioblastoma prognosis remains poor despite a remarkable amount of research programs; thus, this tumor remains a clinical challenge. The high level of inter- and intra-tumor heterogeneity is a major problem in the understanding of the physiopathology of the glioblastoma and requires a fine molecular analysis in order to lead to therapeutic solutions. The work of Tomei S. et al. [12] is part of this challenge. The authors investigated the microRNA profile of glioblastoma stem cells and their role in the progression of glioblastoma and patient outcome. Comparing the differential expression of miRNA in stem cells versus autologous differentiated cells might be of high interest. The authors found some new microRNAs correlated with the patient survival outcome. A review examines the role of the potential use of salivary microRNAs (miRNAs) as diagnostic and prognostic biomarkers for Oral squamous cell carcinoma patients aiding in the establishment of specific therapeutic strategies [13]. Precision and personalized medicine are useful tools for preventive strategies and could help with predicting morbidity and mortality and detecting chronic disease much earlier in the disease course, to improve the quality of care and quality of life of the patients and reduced healthcare time, efforts, and costs. Omics sciences offer a wide range of tools to improve public health including whole-genome and exosome sequencing. In future, disease prevention and treatment should be formulated at the individual level according to genomic features. Omics sciences have important implications for the prevention of both communicable and non-communicable diseases, especially because they can be used to assess health status during the whole course of life [14].
